# Validity of Moyers and Tanaka-Johnston Prediction Methods for Mixed Dentition Analysis in the Pediatric Population of Central Maharashtra: A Cross-Sectional Validation Study

**DOI:** 10.7759/cureus.97549

**Published:** 2025-11-23

**Authors:** Komal Chaudhari, Mayur S Bhattad, Babu GV, Sumit Rajewar, Ankita Chandak, Rohini R Bartakke, Sanpreet S Sachdev

**Affiliations:** 1 Pediatric and Preventive Dentistry, Dr. Hedgewar Smruti Rugna Seva Mandal's Dental College, Hingoli, IND; 2 Pedodontics and Preventive Dentistry, Dr. Hedgewar Smruti Rugna Seva Mandal's Dental College, Hingoli, IND; 3 Pediatric Dentistry, Dr. Hedgewar Smruti Rugna Seva Mandal's Dental College, Hingoli, IND; 4 Oral Pathology and Microbiology, Bharati Vidyapeeth (Deemed to be University) Dental College and Hospital, Navi Mumbai, IND

**Keywords:** dental casts, indian population, mesiodistal width, mixed dentition analysis, moyers method, orthodontics, tanaka–johnston, tooth size prediction

## Abstract

Background

Accurate prediction of the mesiodistal widths of unerupted permanent canines and premolars is essential for mixed-dentition space analysis and orthodontic planning. The Moyers’ probability tables and the Tanaka-Johnston regression equations are widely used, yet their accuracy across different Indian subpopulations remains uncertain.

Aim

To assess and compare the validity of Moyers’ and Tanaka-Johnston's prediction methods for estimating the combined mesiodistal widths of unerupted canines and premolars in children from the Central Maharashtra population.

Methods

This cross-sectional validation study included 40 children aged 6-11 years in mixed dentition. Mesiodistal tooth widths were measured directly on dental casts using a pointed divider and millimeter scale, while the arch perimeter was recorded using 0.5-mm stainless-steel ligature wire. Predicted values were obtained using Moyers’ percentile tables (95th percentile for both sexes) and the Tanaka-Johnston equations. The primary outcome was the mean bias (Available - Predicted, mm). Secondary outcomes included the SD of bias, Bland-Altman limits of agreement, the proportion of predictions within ±1 mm and ±2 mm, and Cohen’s dz. Statistical analyses used paired t-tests and Bland-Altman plots (p < 0.05).

Results

Both methods demonstrated close agreement with actual measurements at the group level. Moyers’ method slightly underestimated mandibular widths by a mean difference of 1.18 mm and nearly matched maxillary values (-0.15 mm). Tanaka-Johnston, on the other hand, showed minimal bias in both arches (≤0.27 mm). Despite non-significant mean differences (all p > 0.05), Bland-Altman limits indicated substantial individual variability.

Conclusion

Moyers’ and Tanaka-Johnston's analyses provide acceptable mean accuracy for Central Maharashtra children but limited reliability for individual predictions. Region-specific regression models are recommended to improve orthodontic diagnostic precision.

## Introduction

Accurate prediction of the mesiodistal widths of unerupted permanent canines and premolars is a cornerstone of effective orthodontic diagnosis and treatment planning during the mixed dentition phase [1,2]. This stage presents a critical window wherein clinicians can intercept potential malocclusions, preserve space, and guide erupting teeth to maintain occlusal harmony [3]. To this end, mixed dentition analysis serves as a non-invasive, practical tool that aids in forecasting future tooth size-arch length relationships, particularly in cases requiring serial extraction or space maintenance [4].

Among the most widely used prediction methods are Moyers’ probability tables and the Tanaka-Johnston regression equations [5,6]. Both approaches are non-radiographic and rely on the mesiodistal width of the four permanent mandibular incisors as predictor variables [7]. Moyers’ analysis provides a range of predicted values based on percentiles, offering flexibility based on clinical judgment, whereas the Tanaka-Johnston equations use fixed mathematical formulae to predict widths for both arches [8-10]. However, it is important to note that both methods were originally developed from data obtained from North American Caucasian populations [11]. Consequently, their applicability in diverse ethnic groups has been increasingly questioned due to well-established racial and ethnic variations in tooth size [12,13].

India, with its vast regional and genetic diversity, presents unique dental anthropometric characteristics that warrant population-specific studies [14]. Previous research has shown variable results regarding the validity of these prediction methods in different Indian subpopulations, indicating a pressing need for localized validation [15,16]. In this context, the Central Maharashtra region remains underrepresented in odontometric research, especially with respect to mixed dentition analyses.

The present cross-sectional validation study was designed to assess the applicability of Moyers’ probability tables and the Tanaka-Johnston regression equations for predicting the mesiodistal widths of unerupted permanent canines and premolars in children from the Central Maharashtra population. All actual tooth widths were measured directly on dental casts using a calibrated divider method, which has been demonstrated to provide reliable estimates comparable to direct intraoral measurements [16].

Therefore, the present study aimed to (1) evaluate the accuracy of the Moyers’ and Tanaka-Johnston methods by comparing predicted and measured combined mesiodistal widths in each arch; (2) quantify the mean prediction bias (measured - predicted) as the primary outcome; (3) determine agreement between methods and true values using Bland-Altman analysis; and (4) explore secondary parameters, including the proportion of cases with an absolute error ≥ 2 mm, arch-length discrepancy, and intra-examiner reliability assessed using the intraclass correlation coefficient (ICC). The results will inform whether existing prediction models are suitable for this regional population or require population-specific recalibration.

## Materials and methods

The present cross-sectional study was conducted in the Department of Pedodontics and Preventive Dentistry over a period of one month, from September 2025 to October 2025. Prior to data collection, ethical clearance was obtained from the Institutional Ethics Committee (Reference Letter No. HDCH/2025/IEC/123, dated 16/09/2025). Informed written consent was obtained from the parents or legal guardians of all participants.

Sample size estimation

The sample size was estimated using the formula:

\[N=\frac{2(Z_{α}+Z_{β})^{2}}{µ_{1}- µ_{2}/σ^{2}}\]

where the denominator is defined as the effect size, while µ_1_ and µ_2_ are the means of the two groups, and σ is the SD of the population being studied. The probability of falsely rejecting a true null hypothesis was 0.2 for the β value, wherein the power is 80% for which the Z_β_ value was 0.84. The level of significance is 5% for which Z_α_ was 1.96. An effect size of 0.90 (Cohen’s d) was adopted based on previously published studies in Indian populations reporting mean differences of approximately 0.8-1.0 mm between predicted and actual mesiodistal tooth widths, corresponding to a large effect size [5,7]. This ensured adequate statistical power (80%) to detect clinically relevant differences between the two prediction methods at a 5% significance level. For an effect size of 0.90, the substituted values yielded a final sample size of 19.75. Therefore, a sample size of 20 was deemed sufficient for a valid statistical comparison between the two methods, making the total sample size required N = 40.

Recruitment of participants

A total of 40 healthy children aged 6 to 11 years, representative of the Central Maharashtra population, were selected for the study. Although 20 subjects were statistically sufficient, 40 children were included to enhance representativeness, allow for potential exclusions due to defective casts, and improve statistical power. All participants were in the mixed dentition phase with fully erupted mandibular permanent incisors and no clinical or radiographic signs of dental anomalies. Inclusion criteria comprised children with intact dentition and no history of orthodontic treatment. Exclusion criteria included hypodontia, supernumerary teeth, grossly carious or restored anterior or premolar teeth, significant attrition, or congenital craniofacial syndromes.

Measurement protocol

Cast Preparation and Calibration

High-quality impressions of both arches were made using irreversible hydrocolloid (Tropicalgin Alginate, Zhermack, Italy) in properly fitting stock trays, ensuring uniform thickness and complete anatomical detail. Each impression was poured immediately in type III dental stone (Kalabhai Kalstone, Mumbai, India) to obtain dimensionally stable casts. All casts were inspected for voids, fractures, or surface defects, and any unsatisfactory models were remade before measurement to prevent distortion-related errors.

The mesiodistal widths of the mandibular permanent incisors and the maxillary and mandibular canines and premolars were measured directly on the dental casts using a pointed divider and a millimeter steel scale (Aerospace Engineers, India) graduated to 0.1 mm. The divider tips were positioned at the greatest mesiodistal contact points of each tooth, ensuring that both points were tangential to the enamel surfaces and aligned parallel to the occlusal plane. The span obtained by the divider was then carefully transferred to the scale for reading. Before every measurement session, the divider was verified against a 10 mm standard gauge line to confirm calibration accuracy. Each tooth was measured three times at separate sittings spaced seven days apart, and the mean of the three readings was recorded as the final value. The examiner was blinded to previous readings and prediction results to minimise bias. All values were recorded to one decimal place using standard half-up rounding. Cast-based measurements obtained with precision dividers have been shown to provide reliable and reproducible results comparable to direct intraoral methods [16].

Arch Perimeter Measurement

**Figure 1:**
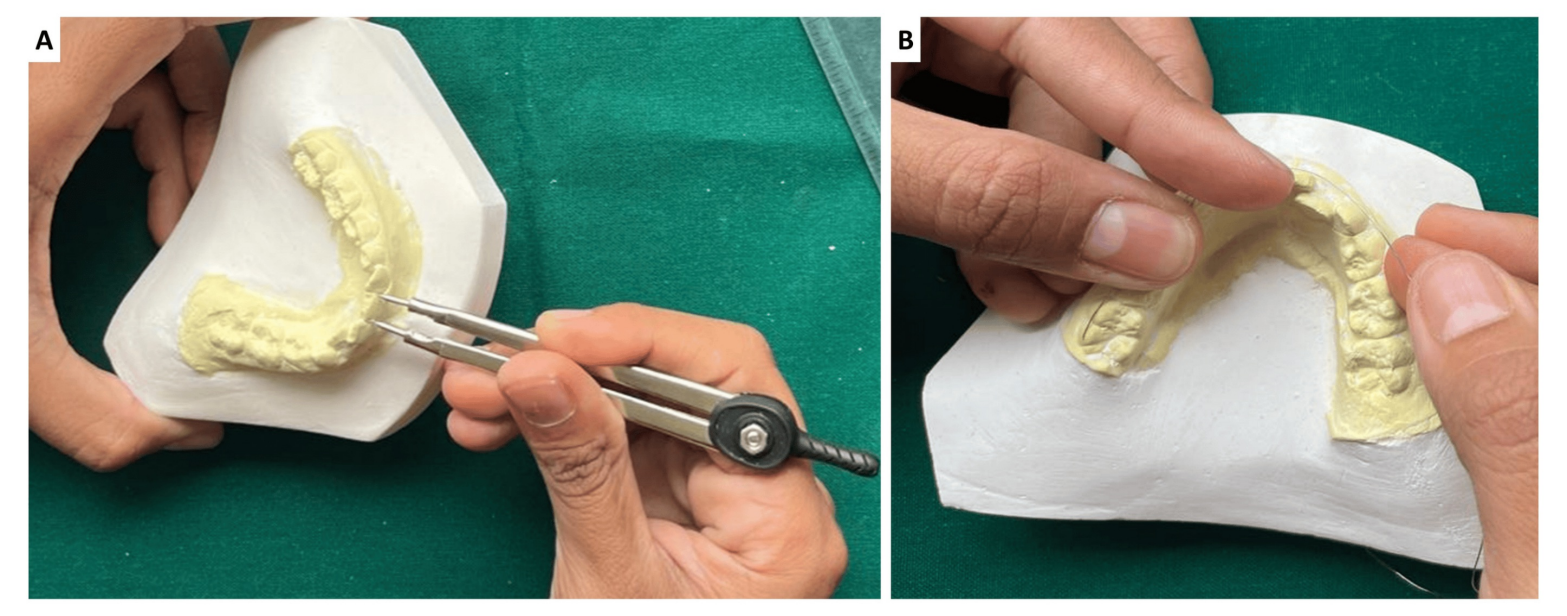
(A) Measurement of the teeth using a pointed divider; (B) Measurement of the arch perimeter using ligature wire.

The available arch perimeter was determined using a 0.5 mm stainless-steel orthodontic ligature wire (G&H Orthodontics, USA). The wire was adapted along the buccal cusp tips and incisal edges from the mesial surface of the right first permanent molar to the mesial surface of the left first permanent molar, following the curvature of the arch without stretching. The wire was then straightened on a glass plate and its length measured using the same divider-and-scale method. This measurement represented the available arch length. All measurements were performed by a single calibrated examiner, whose calibration was confirmed through repeated measurements on five randomly selected casts until an ICC above 0.90 was achieved, indicating excellent agreement (Figure 1).

Analysis using prediction models

For each subject, the sum of the mesiodistal widths of the four mandibular permanent incisors was used as the predictor variable. Predictions of the combined mesiodistal widths of the canines and premolars in each quadrant were made using two approaches: the Tanaka-Johnston analysis and Moyers’ mixed dentition analysis.

The Tanaka-Johnston analysis employs the following equations [17] for the maxilla:

\[Predicted\ width\ of\ maxillary\ premolar\ and\ canine\ in\ each\ quadrant\ =\ 11\ +\ 0.5\ times\ (Sum\ of\ lower\ incisors)\]

and for mandible:

\[Predicted\ width\ of\ mandibular\ premolar\ and\ canine\ in\ each\ quadrant\ =\ 10.5\ +\ 0.5\ times\ (Sum\ of\ lower\ incisors)\]

The table used for Moyers’ mixed dentition analysis is shown in Table 1. It utilizes probability tables at the 35th to 85th percentiles for both maxillary and mandibular arches, separately for males and females [18]. These equations were applied uniformly for both sexes. In Moyers’ mixed dentition analysis, predictions were derived from percentile probability tables calculated separately for each arch and sex. Based on evidence from Indian populations, the 95th percentile was used for both genders, as this level provided optimal predictive accuracy for regional cohorts.

**Table 1:** Moyers’ mixed dentition analysis.

	Maxilla	Mandible
Space Available = Arch Perimeter Analysis	Maxillary Arch Perimeter	Mandibular Arch Perimeter
Space Available = Sum of erupted and unerupted teeth.	Mesiodistal width of maxillary permanent incisors, canines and premolars	Mesiodistal width of mandibular permanent incisors, canines and premolars
Arch length discrepancy	Crowding or spacing	Crowding or spacing

Repeatability and Reliability Assessment

To assess intra-examiner consistency, 10 randomly selected casts, representing 25% of the total sample, were remeasured by the same examiner after a seven-day interval. Repeatability was quantified using Dahlberg’s error, calculated as √(Σd² / 2n), where d denotes the difference between two readings and n is the number of repeated specimens. Reliability was evaluated using ICCs computed with a two-way mixed-effects model, absolute-agreement, single-measure approach (ICC(3,1)). ICC values below 0.50 were interpreted as poor, 0.50-0.75 as moderate, 0.75-0.90 as good, and above 0.90 as excellent reliability.

Outcome Measures and Statistical Plan

The primary outcome of the study was the mean bias between available and predicted combined mesiodistal tooth widths, expressed as (Available - Predicted, mm). Secondary outcomes included the SD of bias, Bland-Altman limits of agreement (mean bias ± 1.96 × SD), the proportion of predictions within ±1.0 mm and ±2.0 mm of the actual measurements, and Cohen’s dz as the paired effect size. All data were entered into Microsoft Excel and analyzed using IBM SPSS Statistics, Version 20.0 (IBM Corp., Armonk, NY, USA). The maxillary and mandibular arches were analyzed independently because the prediction equations and Moyers’ percentiles differ by arch; therefore, no clustering or paired modeling across arches was applied. Normality was assessed with the Shapiro-Wilk test. Comparisons between available and predicted values were performed using the paired t-test for normally distributed datasets and the Wilcoxon signed-rank test when normality was violated. Statistical significance was set at p < 0.05.

Handling of Missing or Unusable Data

Casts that were fractured, distorted, or presented unreadable contact points were excluded from the relevant analyses using pairwise deletion. The number and reason for each exclusion were documented, and no data imputation was performed.

## Results

Participant demographics and characteristics

The mean age of participants was 9.0 ± 1.6 years, comprising an equal number of boys and girls (n = 20 each). All participants were from the Central Maharashtra region, in the mixed dentition stage, with fully erupted mandibular permanent incisors and no dental anomalies or prior orthodontic treatment. Approximately 55% (n = 22) belonged to the 9-11-year subgroup, while 45% (n = 18) were aged 6-8 years. The demographic characteristics of the study population are summarised in Table 2.

**Table 2:** Participant demographics and characteristics (N = 40).

Variable	Category	n (%)
Age (years) mean: 9.0 ± 1.6	6-8	18 (45%)
	9-11	22 (55%)
Gender	Male	20 (50%)
	Female	20 (50%)

Tooth‑size prediction accuracy

Table 3 presents available and predicted mesiodistal widths for each method-arch combination. In the Moyers cohort, the mean available widths were 63.51 mm (mandible) and 67.55 mm (maxilla), while predicted means were 64.69 mm and 67.70 mm, respectively. Tanaka-Johnston yielded available means of 19.80 mm (mandible) and 23.30 mm (maxilla), with predicted means of 19.88 mm and 23.57 mm, respectively. Mean differences in every case were ≤ 1.2 mm, indicating close agreement at the group level, although the standard deviations (SD ≈ 3 mm) confirm substantial individual variability.

**Table 3:** Descriptive statistics of mesiodistal widths (N = 20 per method).

Method × Arch	Available Width (Mean ± SD, mm)	Predicted Width (Mean ± SD, mm)	Mean Difference (mm)
Moyers-Mandible	63.51 ± 5.01	64.69 ± 2.64	-1.18
Moyers-Maxilla	67.55 ± 2.18	67.70 ± 2.15	-0.15
Tanaka-Johnston-Mandible	19.80 ± 3.38	19.88 ± 1.58	-0.08
Tanaka-Johnston-Maxilla	23.30 ± 3.26	23.57 ± 1.10	0.27

Paired comparison of available versus predicted widths

Table 4 shows that none of the paired t-tests reached statistical significance (all p > 0.35). Moyers’ analysis slightly underestimated mandibular widths with a mean difference of 1.18 mm and almost exactly matched maxillary widths (-0.15 mm). Tanaka-Johnston analysis exhibited the smallest mandibular bias (-0.08 mm) and a slight over-prediction of 0.27 mm in the maxilla.

**Table 4:** Paired t-tests: available versus predicted widths.

Group × Arch	t	df	p	Mean Diff (mm)	Conclusion
Moyers-Mandible	-0.929	19	0.359	-1.18	Not significant
Moyers-Maxilla	-0.226	19	0.822	-0.15	Not significant
Tanaka-Johnston-Mandible	-0.09	19	0.929	-0.08	Not significant
Tanaka-Johnston-Maxilla	0.173	19	0.865	+0.27	Not significant

Normality, effect size, and agreement analysis for Tanaka-Johnston method

Shapiro-Wilk testing and paired t-tests (Table 5) confirmed negligible mandibular and maxillary bias, with very small effect sizes (Cohen’s dz = 0.03). Bland-Altman parameters (Table 6) showed minimal mean bias (≤ 0.3 mm) but limits of agreement spanning approximately ± 6 mm. Pearson correlation between available and predicted values was moderate in the mandible (r = 0.48, p = 0.033) and weak in the maxilla (r = 0.21, p = 0.37).

**Table 5:** Normality and paired t-tests for the Tanaka-Johnston model.

Arch	Shapiro W value	Shapiro p-value	Mean Difference (mm)	95% CI	t (df = 19)	p	Cohen’s dz
Mandible	0.935	0.197	0	-1.30 to 1.30	0	1	0
Maxilla	0.883	0.02	0.08	-1.45 to 1.62	0.109	0.915	0.024

**Table 6:** Bland-Altman and Pearson correlation for the Tanaka-Johnston model. LoA: Limits of agreement. ^†^Mean difference (Available - Predicted); ^‡^Limits of agreement: bias ± 1.96 × SD; *p < 0.05.

Arch	Bias† (mm)	SD (mm)	Lower LoA‡ (mm)	Upper LoA‡ (mm)	Pearson r	p
Mandible	–0.08	2.97	–5.90	5.75	0.478	0.033*
Maxilla	–0.28	3.21	–6.57	6.02	0.213	0.368

Arch-length discrepancy in the Moyers analysis

Table 7 summarises the clinical arch-length discrepancy. The mean discrepancy showed a mild, non-significant trend toward mandibular crowding (-0.67 ± 1.78 mm; p = 0.109) and a nearly neutral maxilla (-0.11 ± 2.55 mm; p = 0.982). Crowding occurred in 13 mandibular arches (65%) and 9 maxillary arches (45%), whereas spacing was observed in 6 mandibular arches (30%) and 11 maxillary arches (55%). One mandibular arch (5%) was neutral.

**Table 7:** Arch length discrepancy in Moyers analysis. A negative value denotes crowding; a positive value denotes spacing.

Arch	Mean ± SD (mm)	Range (mm)	t (df = 19)	p
Mandible	-0.67 ± 1.78	-4.6 to +1.6	-1.68	0.109
Maxilla	-0.11 ± 2.55	-3.6 to +8.0	-0.02	0.982

ICC test

Intra-examiner reliability was excellent for all repeated measurements. Dahlberg’s error ranged from 0.055 mm to 0.089 mm for the Moyers assessment and from 0.224 mm to 0.387 mm for the Tanaka-Johnston assessment, indicating very small random measurement error. The ICC ranged from 0.922 to 0.997 for Moyers and from 0.990 to 0.998 for Tanaka-Johnston, confirming excellent reliability according to standard benchmarks (ICC > 0.90 = excellent). All ICC values were statistically significant (p < 0.001). The low Dahlberg errors (< 0.1 mm for Moyers and < 0.4 mm for Tanaka-Johnston) indicate minimal random error, reflecting a high level of examiner precision. ICC values exceeding 0.90 across all variables denote excellent agreement and confirm that the measurement protocol was highly reproducible.

**Table 8:** Dahlberg’s error and reliability analysis for Moyers’ and Tanaka-Johnston analyses. ICC: Intraclass correlation coefficient test.

	Variable	Dahlberg’s error	ICC
Moyer’s analysis	Mesiodistal width of permanent mandibular incisors	0.067	0.922
	Mesiodistal width of mandibular canine and premolar (right)	0.084	0.997
	Mesiodistal width of mandibular canine and premolar (left)	0.087	0.997
	Mesiodistal width of permanent maxillary incisors	0.084	0.996
	Mesiodistal width of maxillary canine and premolar (right)	0.089	0.991
	Mesiodistal width of maxillary canine and premolar (left)	0.055	0.996
Tanaka-Johnston analysis	Available space in mandible	0.224	0.998
	Available space in maxilla	0.387	0.99

## Discussion

The present study evaluated the validity of Moyers’ and Tanaka-Johnston’s prediction methods for estimating the mesiodistal widths of unerupted canines and premolars in an Indian population, using direct measurements from dental casts. Both methods demonstrated close agreement with the actual measurements at the group level; however, substantial individual variability was evident, limiting their predictive reliability in clinical scenarios. In the Moyers cohort, the method underestimated mandibular widths by 1.18 mm and slightly underestimated maxillary widths by 0.15 mm. The Tanaka-Johnston method showed minimal underestimation in the mandible (0.08 mm) and a slightly greater discrepancy in the maxilla (0.27 mm). Although these numerical deviations appear minor, the accompanying SDs (approximately 3 mm) indicate considerable variation in individual values, which may lead to clinically meaningful misjudgments during orthodontic space analysis.

These findings align with those reported by Barkavi P et al. (2024), who evaluated the same prediction methods in a South Indian mixed-dentition cohort and found that Moyers performed better, particularly at the 75th percentile for mandibular arches, while Tanaka-Johnston continued to overestimate in both arches, especially among male subjects [19]. Collectively, these results reinforce the need to recalibrate existing prediction formulas to account for gender and ethnic diversity. In a large-scale North Indian study involving 260 participants, Goyal RK et al. (2014) reported significant overestimation by Moyers at the 75th percentile and smaller but consistent overestimation by Tanaka-Johnston. They addressed these limitations by developing new regression equations suited to the North Indian population, underscoring the inadequacy of universally applied methods in diverse ethnic settings [20]. This trend is further supported by the systematic review by Binu and Ramar (2025), who synthesised data from 20 Indian studies and concluded that both Moyers and Tanaka-Johnston have limited standalone validity across different Indian populations. They emphasised the usefulness of 75th percentile charts over 50th percentile charts but cautioned against clinical application without proper local validation [21]. Patidar D and Patidar DC (2023) similarly recommended percentile-based adjustments, suggesting the use of Moyers’ 75th percentile for Indian males and 50th percentile for females, while ultimately advocating for population-specific formulas [22].

Recent evidence also supports the validity of intraoral and photographic alternatives to traditional cast-based measurements. Alrasheed WA et al. (2022) demonstrated that tooth size-arch length discrepancy, Little’s Irregularity Index, and Bolton’s ratios measured from intraoral or plaster model photographs showed excellent reliability, with intra- and inter-examiner ICCs ranging from 0.85 to 0.99 and negligible differences compared to direct manual measurements [23]. These findings confirm that modern photographic and digital methods can replicate the accuracy of physical model measurements while improving efficiency and patient comfort. However, in the present study, cast-based analysis was selected to maintain methodological uniformity and avoid variability introduced by imaging calibration, though future research may benefit from incorporating validated digital or photographic protocols.

From a clinical standpoint, such prediction inaccuracies can result in misguided interventions. In the current study, mandibular arches showed a mild mean crowding tendency of 0.67 mm, whereas the maxilla demonstrated a nearly neutral discrepancy of 0.11 mm. Overestimation of space requirements, particularly in the maxillary arch using the Tanaka-Johnston method, could lead to unnecessary extractions or orthodontic expansion. Conversely, underestimation may delay timely intervention or compromise final occlusal outcomes [24]. The use of direct intraoral or cast-based measurement tools, as employed in this study, provides an accessible and cost-effective alternative in clinical settings where radiographs are not indicated or feasible [25,26].

A key limitation of the present investigation is its relatively small sample size, which restricts generalizability and statistical robustness. The study did not perform subgroup analyses by gender, despite evidence suggesting sex-based differences in tooth dimensions and prediction accuracy. Additional limitations include reliance on a single examiner without blinding and the absence of comparison with digital measurement techniques, both of which may affect reproducibility and external validity. Although the group-level findings are consistent with previous reports, the small sample size and single-centre design limit the strength of the conclusions. The wide individual variability in prediction accuracy further constrains direct clinical translation. Therefore, these results should be interpreted as preliminary regional evidence supporting the need for population-specific calibration rather than as definitive guidance for treatment planning. Future studies should include larger, demographically diverse cohorts with stratified analyses by age and sex. There is also growing potential for integrating machine learning and artificial intelligence to develop individualized, population-specific prediction tools that could meaningfully enhance diagnostic precision in mixed dentition analysis.

## Conclusions

Within the limits of this cross-sectional validation study, both Moyers’ and Tanaka-Johnston prediction methods demonstrated small mean errors but substantial individual variation in estimating the widths of unerupted teeth among children from Central Maharashtra. Although the average bias was minor (within ±1 mm), the wide limits of agreement (approximately ±6 mm) indicate that predictions for individual patients may vary considerably. The results therefore support cautious clinical application of these methods and suggest that population-specific or digitally recalibrated models may improve predictive accuracy. These findings are applicable only to the studied regional cohort and should not be generalised to other populations or interpreted as evidence of direct clinical impact.
